# Integrating deep learning for post-translational modifications crosstalk on Hsp90 and drug binding

**DOI:** 10.1016/j.jbc.2025.110519

**Published:** 2025-07-25

**Authors:** Jennifer A. Heritz, Katherine A. Meluni, Sarah J. Backe, Sara J. Cayaban, Laura A. Wengert, Meik Kunz, Mark R. Woodford, Dimitra Bourboulia, Mehdi Mollapour

**Affiliations:** 1 Department of Urology, SUNY Upstate Medical University, Syracuse, New York, USA; 2 Department of Biochemistry and Molecular Biology, SUNY Upstate Medical University, Syracuse, New York, USA; 3 Upstate Cancer Center, SUNY Upstate Medical University, Syracuse, New York, USA; 4 The Bioinformatics CRO, Sanford Florida, USA

**Keywords:** Hsp90, chaperone, cochaperone, phosphorylation, acetylation, histone deacetylase, deep learning, artificial intelligence

## Abstract

Post-translational modification (PTM) of proteins regulates cellular proteostasis by expanding protein functional diversity. This naturally leads to increased proteome complexity as a result of PTM crosstalk. Here, we used the molecular chaperone protein, Heat shock protein-90 (Hsp90), which is subject to a plethora of PTMs, to investigate this concept. Hsp90 is at the hub of proteostasis and cellular signaling networks in cancer and is, therefore, an attractive therapeutic target in cancer. We demonstrated that deletion of histone deacetylase 3 (*HDAC3*) and histone deacetylase 8 (*HDAC8*) in human cells led to increased binding of Hsp90 to both ATP and its ATP-competitive inhibitor, Ganetespib. When bound to this inhibitor, Hsp90 from both *HDAC3* and *HDAC8* knock-out human cells exhibited similar PTMs, mainly phosphorylation and acetylation, and created a common proteomic network signature. We used both a deep-learning artificial intelligence (AI) prediction model and data based on mass spectrometry analysis of Hsp90 isolated from the mammalian cells bound to its drugs to decipher PTM crosstalk. The alignment of data from both methods demonstrates that the deep-learning prediction model offers a highly efficient and rapid approach for deciphering PTM crosstalk on complex proteins such as Hsp90.

Heat shock protein-90 (Hsp90) is an essential and evolutionarily conserved molecular chaperone responsible for maintaining cellular proteostasis. Hsp90 achieves this task through involvement in stabilization and activation of more than 300 client proteins (1, 2). The majority of these clients are involved in cellular pathways that are dysregulated in chronic diseases, including cancer (3, 4, 5). Due to its central role in oncogenic pathways, Hsp90 has emerged as a compelling therapeutic target for cancer treatment, with several inhibitors undergoing clinical evaluation (6, 7). Hsp90 chaperone function is associated with its ATPase activity (8). Most Hsp90 inhibitors competitively bind to its ATP-binding pocket, leading to destabilization and degradation of oncogenic client proteins (6, 7). Second-generation ATP-competitive Hsp90 inhibitors, such as Ganetespib (GB) and Pimitespib (TAS-116), have shown promising efficacy in preclinical models and clinical trials, particularly for malignancies, such as lung cancer and gastrointestinal stromal tumors (GIST) (9, 10, 11, 12). The latter has recently been approved for treatment of advanced GIST in Japan (6, 12, 13, 14). Notably, Hsp90 is subject to post-translational modifications (PTMs) that modulate Hsp90 function (15, 16, 17) and can impact its binding to inhibitors (18).

One such PTM, acetylation, is reversed by histone deacetylases (HDACs), which deacetylate Hsp90 and consequently impact its chaperone function (15, 19, 20, 21). Previous studies have also demonstrated that deletion or inhibition of specific HDACs enhances the sensitivity of cancer cells to Hsp90 inhibitors by increasing the acetylation of key lysine residues, thereby promoting more stable drug interactions (22, 23). Recent work has shown that dual-function inhibitors, such as MPT0G449, which simultaneously target histone deacetylases (HDACs) and Hsp90, cause disruption of key cancer-driving pathways with potent antitumor activity in models such as acute leukemia (23).

Despite these insights, the interplay between different PTMs on Hsp90 and their collective impact on drug binding remains poorly understood. Based on previously published work, there is a strong link between Hsp90 binding to its inhibitors and the sensitivity of cells to these inhibitors (15, 17, 24, 25). We applied a systematic approach to determine the impact of each HDAC on Hsp90 binding to its biotinylated second-generation inhibitor, biotinylated-Ganetespib (bio-GB). Deletion of *HDAC3* or *HDAC8* in human cells enhanced Hsp90 binding to ATP and its inhibitor GB. Hsp90 bound to GB from these HDAC KO cells displayed a distinct protein interaction network and PTM pattern from WT cells.

By integrating mass spectrometry with a deep-learning AI model, our study successfully mapped PTM crosstalk on Hsp90, revealing that AI-based prediction closely aligns with experimental data. These findings not only uncover a mechanistic link between HDAC activity and Hsp90 function but also highlight the utility of AI-driven tools for predicting PTM dynamics. It also offers a scalable platform for future studies of complex protein regulation and drug response.

## Results

### Loss of *HDAC3* or *HDAC8* impacts Hsp90 binding to drug and ATP

**Figure 1:**
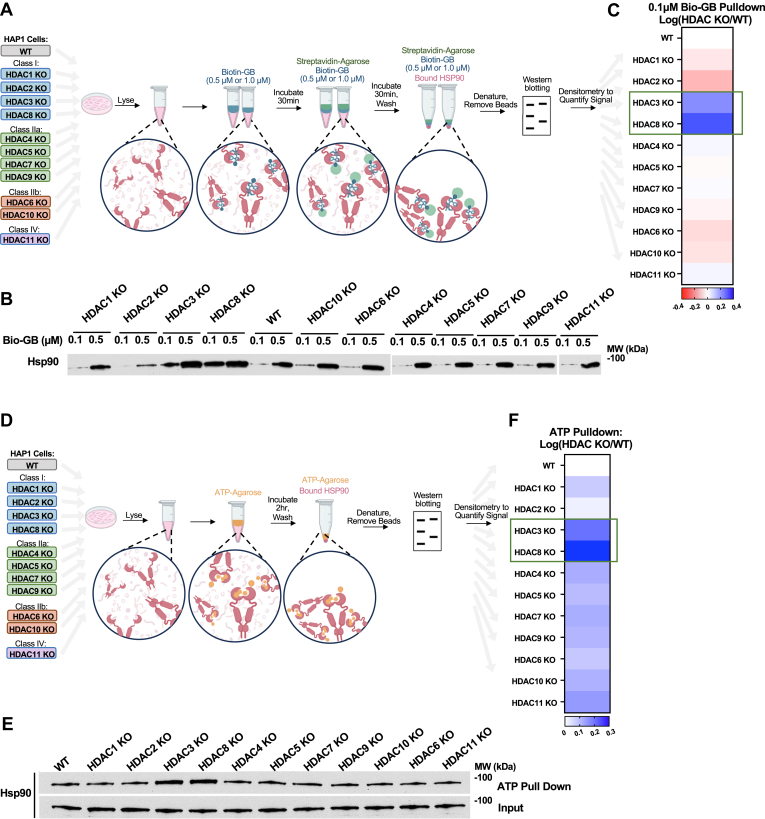
**Loss of *HDAC3* or *HDAC8* impacts Hsp90 binding to drug and ATP**. *A*, schematic representation of Biotin-GB pulldown workflow. HAP1 WT or HAP1 HDAC KO cell lysate was collected and incubated with Biotin-GB (0.1 μM or 0.5 μM) for 30 min. Streptavidin beads were then added and incubated for an additional 30 min. Proteins were eluted from beads and run on an SDS-PAGE gel for immunoblot analysis. Densitometry was used to quantify Hsp90 binding to Biotin-GB. See also Fig. S1, *A*–*C*. *B*, HAP1 WT or HDAC1-11 KO cell lysate were challenged with 0.1 μM or 0.5 μM Biotin-GB (Bio-GB). Hsp90 binding to Bio-GB was assessed by immunoblotting. Representative blot of three independent experiments; see also Fig. S1*B*. *C*, heatmap of densitometry values from Figures 1, *A* and *B* and S1*B* quantifying Hsp90 binding to 0.1 μM Biotin-GB. Data displayed is log(fold-change) intensity of the Hsp90 bound to 0.1 μM Biotin-GB in each HAP1 HDAC KO relative to that in HAP1 WT. *Red* shading indicates decreased binding of Hsp90 to Biotin-GB whereas blue shading indicates increased binding. To ensure accurate quantifications, Hsp90 pulldown values were normalized to Hsp90 input values for each sample. See also Fig. S1*B*. *D*, schematic representation of ATP pulldown workflow. HAP1 WT or HAP1 HDAC KO cell lysate was collected and incubated with ATP-agarose for 2 h. Proteins were eluted from beads and run on SDS-PAGE gel for immunoblot analysis. Densitometry was used to quantify Hsp90 binding to ATP. See also Fig. S1*D*. *E*, HAP1 WT or HDAC1-11 KO cell lysate were challenged with ATP-agarose. Hsp90 binding to ATP-agarose was assessed by immunoblotting. Representative blot of three independent experiments; see also Fig. S1*D*. *F*, heatmap of densitometry values from Figures 1, *D* and *E* and S1*D* quantifying Hsp90 binding to ATP-Agarose. Data displayed is log(fold-change) intensity of the Hsp90 bound to ATP-Agarose in each HAP1 HDAC KO relative to Hsp90 bound to ATP-Agarose in HAP1 WT. *Blue* shading indicates increased binding of Hsp90 to ATP-Agarose. To ensure accurate quantifications, Hsp90 pulldown values were normalized to Hsp90 input values for each sample. See also Fig. S1*D*.

It is generally accepted that Hsp90 binding to its inhibitors correlates with cell sensitivity to these inhibitors (24); therefore, we applied a systematic approach to determine Hsp90 binding to its second-generation inhibitor, Ganetespib (GB). Biotinylated-GB (bio-GB) at 0.1 μM or 0.5 μM were used to challenge cell lysate from wild-type (WT) HAP1 cells and HAP1 cells with each HDAC gene individually knocked-out (KO) (*HDAC1* through *HDAC11*) (Figs. 1, *A*–*C* and S1, *A*–*C*). HAP1 cells are a haploid human cell line derived from the KBM-7 chronic myelogenous leukemia (CML) cell line (26). Hsp90 binding to bio-GB was assessed by immunoblotting and revealed that deletion of *HDAC3* or *HDAC8* increased Hsp90 binding to bio-GB (Figs. 1, *A*–*C* and S1, *A*–*C*). Since Hsp90 chaperone function is dependent on its ability to bind and hydrolyze ATP, and since Ganetespib binds to the ATP binding pocket of Hsp90, we conducted similar experiments using ATP agarose. Our data demonstrated most of the HDAC KOs caused an increase in Hsp90 binding to ATP, however, Hsp90 from *HDAC8* KO cells displayed the highest binding to ATP (Figs. 1, *D* and *E* and S1*D*). Collectively, our qualitative data presented here revealed that deletion of *HDAC3* or *HDAC8* enhanced Hsp90 binding to its inhibitor and ATP.

### Deletion of *HDAC3* or *HDAC8* differentially dictates global interactome of Hsp90 bound to drug

**Figure 2:**
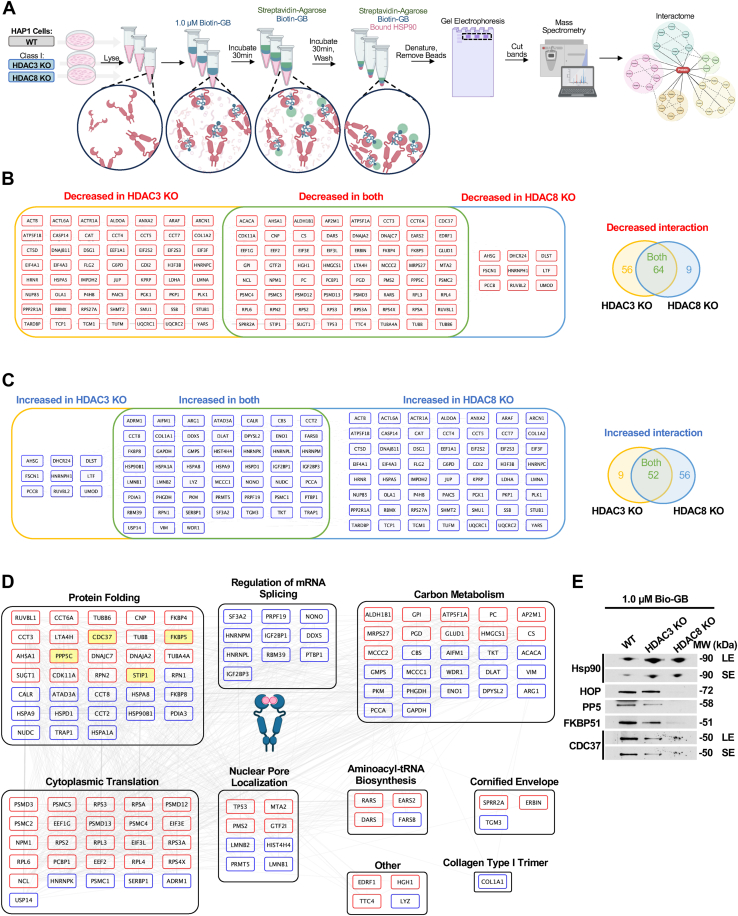
**Deletion of *HDAC3* or *HDAC8* differentially dictates global interactome of Hsp90 bound to drug**. *A*, schematic representation of experimental workflow. HAP1 WT or HAP1 *HDAC3* or *HDAC8* KO cell lysate was collected and incubated with Biotin-GB (0.1 μM) for 30 min. Streptavidin beads were then added and incubated for an additional 30 min. Proteins were eluted from beads and run on SDS-PAGE gel. Bands were excised and subjected to Mass Spectrometry to identify proteins bound to Hsp90-bio-GB complex. *B*, proteins that displayed decreased interaction with Hsp90-bio-GB complex in either *HDAC3* KO or *HDAC8* KO relative to WT. Venn diagram of number of proteins with decreased binding to Hsp90-bio-GB complex in only the *HDAC3* KO sample, only the *HDAC8* sample, or both samples relative to WT HAP1. Related to Fig. S2*A*, boxes 1–3. *C*, proteins that displayed increased interaction with Hsp90-bio-GB complex in either *HDAC3* KO or *HDAC8* KO relative to WT. Venn diagram of number of proteins with increased binding to Hsp90-bio-GB complex in only the *HDAC3* KO sample, only the *HDAC8* sample, or both samples relative to WT HAP1. Related to Fig. S2*A*, boxes 1–3. *D*, proteins that displayed a similar binding pattern in both *HDAC3* and *HDAC8* KO cells were analyzed for gene ontology biological process enrichment. *Red* indicates proteins with decreased interaction with Hsp90-bio-GB complex in both *HDAC3* and *HDAC8* KO cells relative to WT HAP1 cells. *Blue* indicates proteins with increased interaction with Hsp90-bio-GB complex in both *HDAC3* and *HDAC8* KO cells relative to WT HAP1 cells. Proteins displayed based on the groupings from string.db. *E*, HAP1 WT, *HDAC3* KO, and *HDAC8* KO cell lysate were challenged with 1 μM Biotin-GB (Bio-GB). Proteins identified in 2B to have decreased interaction with Hsp90-bio-GB complex were assessed by immunoblotting.

The molecular chaperone Hsp90 has more than 300 client proteins as well as approximately 20 to 25 co-chaperones (27). Post-translational modifications (PTMs) impact the binding of these proteins to Hsp90. Although previous work has focused on Hsp90 interaction with a few of these proteins in a cellular context, limited studies have set out to explore the population of clients and co-chaperones bound to Hsp90 in complex with its inhibitor. We set out to explore the Hsp90-bio-GB complex global interactome from *HDAC3* or *HDAC8* KO cells. We challenged HAP1 wild-type (WT) cells as well as *HDAC3* and *HDAC8* KO cell lysates with 1.0 μM bio-GB followed by mass-spectrometry analysis (see EXPERIMENTAL PROCEDURES, Figs. 2*A* and S2, *A*–*C*). Our data revealed reduced interaction in 64 proteins to Hsp90-bio-GB complex from both *HDAC3* and *HDAC8* KO cells compared to WT (Fig. 2*B*). Conversely, we identified 52 proteins bound strongly to the Hsp90-bio-GB complex from both *HDAC3* and *HDAC8* KO cells (Fig. 2*C*). Further analysis of the 64 proteins with decreased binding in both KOs and 52 proteins with increased binding in both KOs identified various components of cellular pathways, such as cytoplasmic translation and protein folding, suggesting potential alteration of these pathways as a consequence of Hsp90 binding to GB (Fig. 2*D*). Notably, we observed a reduction in HOP (*STIP1*), CDC37 (*CDC37*), PP5 (*PPP5C*), and FKBP51 (*FKBP5*) co-chaperones binding to Hsp90-bio-GB from both *HDAC3* and *HDAC8* KO cells. We confirmed the dissociation of these co-chaperones with Hsp90-bio-GB by immunoblotting (Fig. 2*E* and S2*D*). This is in line with previously published work that the absence of HOP increases Hsp90 binding to its inhibitors (28, 29). These findings suggest that altered PTMs in HDAC-deficient cells not only affect Hsp90 conformation but also reprogram its interactome in the presence of inhibitors.

### Hyperacetylation of Hsp90α-K283 and Hsp90β-K275 enhances drug binding

**Figure 3:**
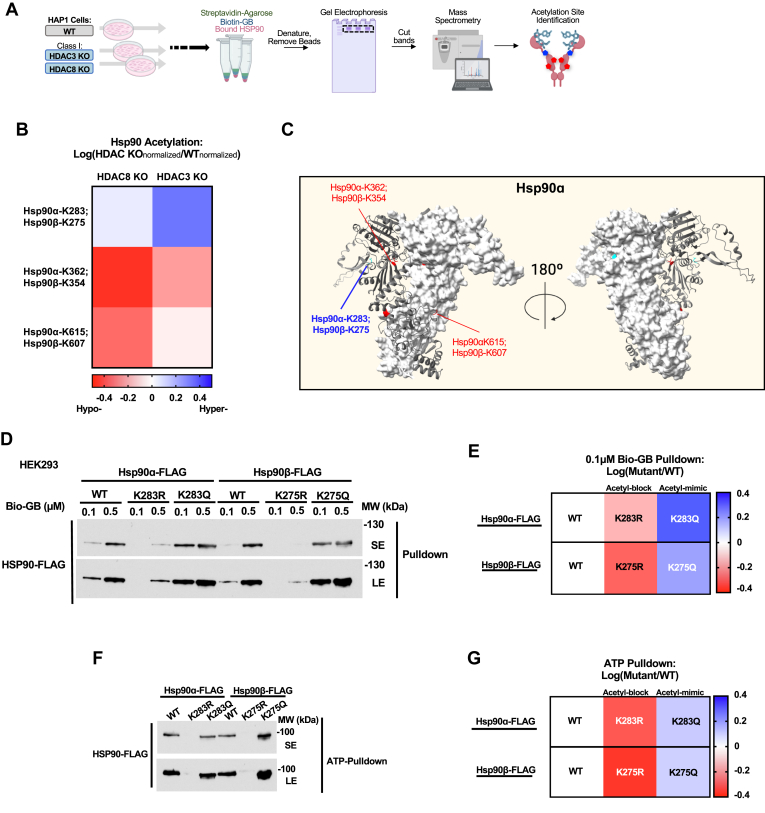
**Hyperacetylation of Hsp90α-K283 and Hsp90β-K275 enhances drug binding.***A*, schematic representation of experimental workflow. HAP1 WT or HAP1 *HDAC3* or *HDAC8* KO cell lysate was collected and incubated with Biotin-GB (0.1 μM) for 30 min. Streptavidin beads were then added and incubated for an additional 30 min (see Fig. 2*A*). Proteins were eluted from beads and run on SDS-PAGE gel. Bands were excised and subjected to mass spectrometry to identify acetylated lysine residues of Hsp90. *B*, Heatmap of densitometry values from Figure 3*A* quantifying Hsp90 acetylation identified by mass spectrometry. Data displayed is log(fold-change) of the intensity of acetylated Hsp90 residue in each HAP1 HDAC KO cells relative to the intensity of acetylated Hsp90 residue in HAP1 WT cells. *Red* indicates hypoacetylation in *HDAC3* or *HDAC8* KO Hsp90-bio-GB complex relative to WT cells, whereas *blue* indicates hyperacetylation in *HDAC3* or *HDAC8* KO relative to WT cells. WT and *HDAC3* or *HDAC8 KO* values were normalized using protein abundance. For peptides which mass spectrometry could not discriminated between Hsp90α and Hsp90β, the residue number for each isoform is displayed. Related to Fig. S2*A*, boxes 4–6. *C*, Hsp90 acetylated lysine sites highlighted on Hsp90α Alphafold structure (amino acids 14–686 shown). Residues that could not be discerned to be either Hsp90α or Hsp90β are separated by a semi-colon. Residues shown in *red* are hypoacetylated. Residues shown in *blue/cyan* are hyperacetylated. *D*, HEK293 cell lysate expressing WT-Hsp90-FLAG or acetylation-blocking (R) or acetylation-mimicking (Q) mutants were challenged with 0.1 μM or 0.5 μM Biotin-GB (Bio-GB). Hsp90 binding to Bio-GB was assessed by immunoblotting. Representative blot of three independent experiments; see also Fig. S3, *C* and *D*. *E*, Heatmap of densitometry values quantifying WT-Hsp90-FLAG or acetylation-blocking (R) or acetylation-mimicking (Q) mutants binding to 0.1 μM Biotin-GB. Hsp90 constructs were transiently expressed in HEK293 cells and Hsp90 bound to GB was isolated from lysate and assessed by immunoblot. Data displayed is log(fold-change) intensity of each Hsp90-FLAG construct bound to 0.1 μM Biotin-GB relative to WT-Hsp90-FLAG bound to 0.1 μM Biotin-GB. *Red* shading indicates decreased binding of the Hsp90 construct to Biotin-GB whereas *blue* shading indicates increased binding of the Hsp90 construct to Biotin-GB. To ensure accurate quantifications, Hsp90-FLAG pulldown values were normalized to Hsp90-FLAG input values for each sample. See also Fig. S3, *C* and *D*. *F*, HEK293 cell lysate expressing WT-Hsp90-FLAG or acetylation-blocking (R) or acetylation-mimicking (Q) mutants were challenged with ATP-agarose. Hsp90 binding to ATP-agarose was assessed by immunoblotting. Representative blot of three independent experiments; see also Fig. S3*F*. *G*, Heatmap of densitometry values from quantifying WT-Hsp90-FLAG or acetylation-blocking (R) or acetylation-mimicking (Q) mutants binding to ATP-Agarose. Hsp90 constructs were transiently expressed in HEK293 cells (as in Fig. 3*D*). Cell lysate was incubated with ATP-agarose and Hsp90 binding to ATP was assessed by immunoblotting. Data displayed is log(fold-change) intensity of each Hsp90-FLAG construct bound to ATP-Agarose relative to WT-Hsp90-FLAG bound to ATP-Agarose. *Red* shading indicates decreased binding of the Hsp90 construct to ATP-Agarose whereas *blue* shading indicates increased binding of the Hsp90 construct to ATP-Agarose. To ensure accurate quantifications, Hsp90-FLAG pulldown values were normalized to Hsp90-FLAG input values for each sample. See also Fig. S3, *C* and *F*.

It is well-established that PTMs of Hsp90 impact drug binding and enhance sensitivity of cells to Hsp90 inhibitors (15, 18). Here, we set out to identify acetylation sites that impact Hsp90 binding to drugs from *HDAC3* or *HDAC8* KO cells. Our data obtained by mass-spectrometry analysis of Hsp90-bio-GB complex isolated from these cells identified hyperacetylation of Hsp90α-K283, or Hsp90β-K275 (Figs. 3, *A* and *B*, S2*A*, and S3*A*). Additionally, we identified hypoacetylation of Hsp90α-K362 or Hsp90β-K354, and Hsp90α-K615 or Hsp90β-K607 (Figs. 3, *B* and *C* and S3, *A* and *B*). Subsequently, we mutated Hsp90α-K283 and Hsp90β-K275 to non-acetylated arginine (R) or acetylation-mimetic glutamine (Q). We next transiently expressed these mutants in HEK293 cells, followed by challenging the lysate with 0.1 μM or 0.5 μM bio-GB. Our results revealed that acetylation-mimetic mutants exhibited a similar increase on Hsp90 drug binding to *HDAC3* or *HDAC8* KO cells (Figs. 3, *D* and *E* and S3, *C*–*E*). Conversely, non-acetylated Hsp90 mutants exhibited decreased binding to bio-GB (Figs. 3, *D* and *E* and S3, *C*–*E*). Similarly, when lysate was challenged with ATP agarose beads, we obtained increased binding in the acetylation-mimetic Hsp90 mutants and decreased binding in the non-acetylated Hsp90 mutants (Figs. 3, *F* and *G* and S3*F*). Collectively, our data revealed that hyper-acetylation of specific lysine residues in Hsp90 modulates its binding to inhibitors.

### Predicting PTM crosstalk of Hsp90 using a deep learning AI model

**Figure 4:**
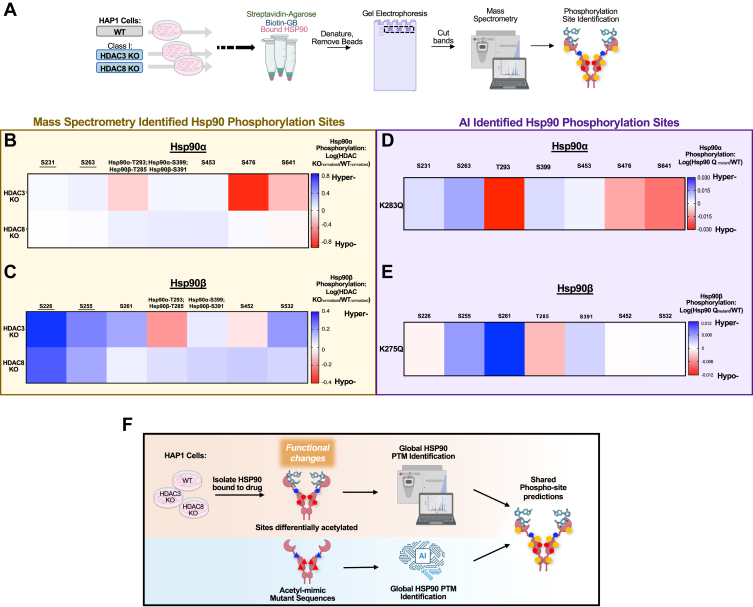
**Predicting PTM crosstalk of Hsp90 using a deep learning AI model**. *A*, schematic representation of experimental workflow. HAP1 WT or HAP1 *HDAC3* or *HDAC8* KO cell lysate was collected and incubated with Biotin-GB (0.1 μM) for 30 min. Streptavidin beads were then added and incubated for an additional 30 min (see Fig. 2*A*). Proteins were eluted from beads and run on SDS-PAGE gel. Bands were excised and subjected to mass spectrometry to identify phosphorylation of residues of the Hsp90-bio-GB complex. Related to Fig. S2*A*, boxes 4–6. *B*, heatmap of densitometry values from Figure 4*A* quantifying Hsp90α phosphorylation identified by mass spectrometry. Data displayed is log(fold-change) of the intensity of phosphorylated Hsp90α residue in each HAP1 HDAC KO relative to the intensity of phosphorylated Hsp90α residue in HAP1 WT cells. *Red* indicates hypo-phosphorylation in *HDAC3* or *HDAC8* KO Hsp90α-bio-GB complex relative to WT cells, whereas blue indicates hyper-phosphorylation in *HDAC3* or *HDAC8* KO relative to WT cells. WT and *HDAC3* or *HDAC8 KO* values were normalized using protein abundance. For peptides which mass spectrometry could not discriminated between Hsp90α and Hsp90β, the residue number for each isoform is displayed. Related to Fig. S2*A*, boxes 4–6. *C*, heatmap of densitometry values from Figure 4*A* quantifying Hsp90β phosphorylation identified by mass spectrometry. Data displayed is log(fold-change) of the intensity of phosphorylated Hsp90β residue in each HAP1 HDAC KO relative to the intensity of phosphorylated Hsp90β residue in HAP1 WT cells. *Red* indicates hypo-phosphorylation in *HDAC3* or *HDAC8* KO Hsp90β-bio-GB complex relative to WT cells, whereas *blue* indicates hyper-phosphorylation in *HDAC3* or *HDAC8* KO relative to WT cells. WT and *HDAC3* or *HDAC8 KO* values were normalized using protein abundance. For peptides which mass spectrometry could not discriminated between Hsp90α and Hsp90β, the residue number for each isoform is displayed. Related to Fig. S2*A*, boxes 4–6. *D*, heatmap of predicted Hsp90α phosphorylation intensities identified by the deep-learning artificial intelligence (AI) prediction model adapted from MIND-S and trained with Hsp90α-WT and Hsp90α-K283Q sequences. Data displayed is log(fold-change) of the predicted intensity of phosphorylated residues on Hsp90α-K283Q relative to the predicted intensity of phosphorylated residues on WT Hsp90α. *Red* indicates hypo-phosphorylation of phosphorylated residues on Hsp90α-K283Q relative to WT Hsp90α, whereas *blue* indicates hyper-phosphorylation of phosphorylated residues on Hsp90α-K283Q relative to WT Hsp90α. For peptides which mass spectrometry could not discriminated between Hsp90α and Hsp90β, the residue number for each isoform is displayed. *E*, heatmap of predicted Hsp90β phosphorylation intensities identified by the deep-learning artificial intelligence (AI) prediction model adapted from MIND-S and trained with Hsp90β-WT and Hsp90β-K275Q sequences. Data displayed is log(fold-change) of the predicted intensity of phosphorylated residues on Hsp90β-K275Q relative to the predicted intensity of phosphorylated residues on WT Hsp90β. Red indicates hypo-phosphorylation of phosphorylated residues on Hsp90β-K275Q relative to WT Hsp90β, whereas blue indicates hyper-phosphorylation of phosphorylated residues on Hsp90β-K275Q relative to WT Hsp90β. For peptides which mass spectrometry could not discriminated between Hsp90α and Hsp90β, the residue number for each isoform is displayed. *F*, a deep-learning artificial intelligence (AI) model can accurately predict the effect of differential acetylation on the global PTM landscape of Hsp90. Hsp90 bound to drug isolated from WT, *HDAC3*, or *HDAC8* KO cells were found to be differentially acetylated, leading to functional changes. Mass spectrometry analysis revealed that this population of Hsp90 bound to drug also has differentially phosphorylated sites. Using acetyl-mimetic mutant sequences of Hsp90 at these residues as an input, the AI model identified the same differentially phosphorylated residues as were determined experimentally.

Although hyperacetylation of Hsp90 enhanced its drug and ATP binding, it is unclear whether this modification impacted other PTMs, such as phosphorylation. We first isolated Hsp90 in complex with bio-GB from *HDAC3* or *HDAC8* KO and WT HAP1 cells. We next identified phosphorylation sites on these populations of Hsp90 bound to bio-GB by mass-spectrometry (Figs. 4*A* and S4*A*). Our data revealed hyperphosphorylation sites of Hsp90α-S231, -S263, -S399, and -S453 (Fig. 4*B*). Hyperphosphorylation sites were also identified in Hsp90β-S226, -S255, -S261, -S391, and -S532 (Fig. 4*C*). The residue number for each isoform is displayed for peptides which mass spectrometry could not discriminate between Hsp90α and Hsp90β (Fig. 4, *B* and *C*). We next utilized the power of deep learning AI as a predictive means to determine the impact of acetylation of Hsp90 toward its global phosphorylation. We used a similar strategy as in previously published work to design a specific AI model for PTM prediction on Hsp90 (30). We created a model based on the multi-label interpretable deep-learning method for PTM prediction-structure version (MIND-S) framework (30, 31) to predict PTMs in Hsp90, fine-tuning it using curated WT and mutant sequences (see EXPERIMENTAL PROCEDURES). Given that our data identified that Hsp90α-K283 is hyperacetylated in both *HDAC3* or *HDAC8* KO cells (Fig. 3*B*), we changed Hsp90α-K283 and Hsp90β-K275 to acetylation-mimetic glutamine (Q) and analyzed the protein sequence by our AI-based tool. The results predicted hyperphosphorylation of similar sites as we identified by mass-spectrometry analysis. Our AI model confirmed similar trends in hyper- and hypo-phosphorylation of Hsp90α S231, S263, S399, S453, S476, and S641 and Hsp90β S255, S261, S391, and S532 to our data generated from *HDAC3* or *HDAC8* KO mass-spectrometry (Fig. 4, *D* and *E*). Notably, mass-spectrometry revealed different patterns of phosphorylation on Hsp90α-T293;Hsp90β-T285, Hsp90α-S476, and Hsp90β-S452 between *HDAC3* and *HDAC8* KO (Fig. 4, *B*–*E*). In addition, the limitation of mass spectrometry being unable to differentiate certain peptides between Hsp90 isoforms was overcome by the AI model, resulting in unique scores for each PTM within each isoform (Fig. 4, *D* and *E*). Taken together, our data obtained from deep learning AI is in agreement with phosphorylation trends that were identified by mass-spectrometry of Hsp90-bio-GB complex from *HDAC3* or *HDAC8* KO cells (Fig. 4*F*). Furthermore, our AI tool designed specifically for Hsp90 PTMs can serve as a useful resource to predict PTM crosstalk on Hsp90.

## Discussion

Heat shock protein-90 (Hsp90) remains an attractive and clinically relevant target for cancer therapy due to its role in stabilization and activation of numerous oncogenic client proteins. Several ATP-competitive inhibitors of Hsp90, including Ganetespib (GB) and Pimitespib (TAS-116), have shown efficacy in preclinical and clinical settings (6). The latter has recently been approved for treatment of advanced gastrointestinal stromal tumor (GIST) in Japan (6, 12, 13, 14). Post-translational modifications of Hsp90, particularly acetylation and phosphorylation, are increasingly recognized as critical modulators of its conformation, activity, and drug-binding affinity (15, 17, 24, 25). Our study highlights how the loss of *HDAC3* or *HDAC8* enhances Hsp90 binding to both ATP and its inhibitor, GB (Fig. 4*F*).

Our mass spectrometry of Hsp90-GB complexes identified distinct sets of proteins with either enhanced or reduced binding in both *HDAC3* or *HDAC8* KO backgrounds compared to wild-type cells. Notably, 52 proteins exhibited increased association, while 64 showed decreased interaction, suggesting that HDAC-regulated PTMs reshape Hsp90 interaction landscape. These changes encompassed key pathways involved in protein folding and cytoplasmic translation. Importantly, the co-chaperone HOP (STIP1), known to regulate client loading to Hsp70, was reduced in both *HDAC3* and *HDAC8* KO cells, aligning with previous reports linking HOP depletion to enhanced Hsp90 inhibitor binding (28, 29). These findings suggest that altered PTMs in HDAC-deficient cells not only affect Hsp90 conformation but also reprogram its interactome in the presence of inhibitors (Fig. 2*D*). Of note, based on the presented data, we cannot discriminate whether these changes reflect interaction strength between Hsp90 and its binding partners *versus* changes in binding partner protein abundance. However, given our panel of confirmed interactions demonstrates that the total protein abundance was unchanged (Figs. 2*E* and S2*D*), we expect that these changes are due to changes in binding partner strength.

Our mass spectrometry-based profiling also revealed that Hsp90 from *HDAC3* or *HDAC8* knock-out cells shared similar PTM signatures, prominently featuring acetylation at Hsp90α-K283; Hsp90β-K275. Functional validation confirmed that acetylation-mimetic mutations at these sites enhanced drug binding, whereas non-acetylatable mutants exhibited reduced interaction. Collectively, our findings support a model in which HDAC-mediated regulation of lysine acetylation potentially controls Hsp90 conformational states that favor its interaction with drugs. We further show that acetylation is not an isolated event. Instead, it coordinates with phosphorylation to produce a broader PTM landscape that likely drives client and co-chaperone protein interactions and drug responsiveness. Deletion of *HDAC3* or *HDAC8* in human cells resulted in consistent hyperphosphorylation patterns on several serine residues of both Hsp90α and Hsp90β. This suggests a crosstalk mechanism, where acetylation at a specific lysine residue influences the phosphorylation of adjacent or distant serine and threonine sites. Understanding these interdependencies is crucial for deciphering how PTMs cooperate to regulate Hsp90 function in cancer.

To address the complexity of PTMs crosstalk, we developed a deep learning AI model tailored to predict PTMs on Hsp90. The model was based on the MIND-S framework (30, 31) and trained using curated Hsp90 sequences and our mass spectrometry datasets. We tested its ability to predict the phosphorylation consequences of lysine acetylation using acetylation-mimetic Hsp90 sequences. The AI-predicted phosphorylation patterns closely matched those observed experimentally (*i.e.* by mass-spectrometry). This alignment not only validates the accuracy of our AI platform but also demonstrates its utility in modeling PTM crosstalk.

The integration of AI and proteomics presents a scalable approach to predict how one PTM may influence another in a context-dependent manner. This is particularly relevant for proteins like Hsp90 that are heavily post-translationally modified. Our AI framework offers a rapid and resource-efficient tool to predict PTM patterns, which can inform therapeutic strategies, guide mutant design, and prioritize PTM targets for experimental validation. In summary, our work uncovers a novel mechanism by which *HDAC3* and *HDAC8* regulate Hsp90 through coordinated acetylation and phosphorylation. Our findings highlight how PTMs impact drug binding and suggest that modulating these modifications can enhance the efficacy of Hsp90 inhibitors. Furthermore, the development of a deep-learning model for PTM crosstalk prediction provides a powerful resource for future investigations into Hsp90 biology and the broader landscape of PTM-regulated proteins in cancer.

## Experimental procedures

### Reagents and chemicals

#### Antibodies

Antibodies for HDAC1-8 (5356, 5113, 85057, 7628, 20458, 7612, 33418) and HDAC11 (66042), GAPDH (97166), and all secondary antibodies raised in mouse (7076), rabbit (7074), and rat (7077) were purchased from Cell Signaling Technologies. Antibodies for HDAC9-10 (ab109446, ab108934) were purchased from Abcam. A pan Hsp90 monoclonal antibody (16F1) was purchased from Enzo. An anti-FLAG (DYKDDDDK) tag polyclonal antibody (PA1-984B) was purchased from ThermoFisher Scientific.

#### Bacterial and viral strains

DH5-alpha electrocompetent *E. coli* cells (CC-203) were purchased from Gold Biotechnology.

#### Chemicals, peptides, and recombinant proteins

Biotin-Ganetespib (STA-12-7346) was purchased from Synta Pharmaceuticals.

#### Critical commercial assays

Mirus TransIT-2020 (MIR5405) was purchased from MirusBio. ATP agarose beads (ab270535) were purchased from Abcam. Streptavadin agarose beads (PI20349) were purchased from ThermoFisher Scientific.

#### Deposited data

Raw and analyzed data from this paper were deposited into the ProteomeXchange Consortium *via* the PRIDE (PXD061927) (32). Code for this paper was deposited at Mendeley Data Digital Commons and is publicly available at the time of publication at https://doi.org/10.17632/dk8yb2prrw.1.

#### Cell lines

HEK293 cell line (CRL-1573) was purchased from ATCC (Manassas, Virginia). HAP1 WT and HDAC1-11 KO (C631, HZGHC004120c002, HZGHC001012c006, HZGHC000579c003, HZGHC001011c011, HZGHC001010c007, HZGHC004106c010, HZGHC001016c001, HZGHC001015c010, HZGHC001014c011, HZGHC001003c005, HZGHC001007c002) cell lines were purchased from Horizon Discovery (Cambridge, UK). Cultured human embryonic kidney (HEK293) cells were grown in Dulbecco’s Modified Eagle Medium (DMEM, Sigma-Aldrich), parental (WT) and *HDAC* KO HAP1 cells were cultured in Isocove’s Modified Dulbecco’s Medium (IMDM; Gibco). All growth media were supplemented with 10% fetal bovine serum (FBS, Sigma-Aldrich). HEK293 cells were acquired from (American Type Culture Collection, ATCC), and HAP1 cells were purchased from Horizon Discovery. Cells were maintained in a CellQ incubator (Panasonic Healthcare) at 37 °C in an atmosphere containing 5% CO_2_.

#### Plasmids

Hsp90α-FLAG, Hsp90β-FLAG were reported previously (33). Point mutations were synthesized by GenScript and confirmed by DNA sequencing.

#### Recombinant DNA

pcDNA3-Hsp90α-FLAG and pcDNA3-Hsp90β-FLAG plasmids used were previously published under (33). Point mutations (pcDNA3-Hsp90α-FLAG-K293 R, pcDNA3-Hsp90α-FLAG-K293Q, pcDNA3-Hsp90β-FLAG-K275R, and pcDNA3-Hsp90β-FLAG-K275Q) were synthesized by Genscript.

#### Mass spectrometry

Isolated proteins from the pulldown experiments were subjected to SDS-PAGE gel, and following Coomassie staining, visible bands were manually cut into small pieces approximately 1 mm × 1 mm. The selected protein gel bands were digested in-gel with trypsin overnight at 37 °C following reduction with dithiothreitol (DTT) and alkylation with iodoacetamide at the Weill Cornell Medicine Meyer Cancer Center Proteomics& Metabolomics Core Facility. The resulting peptides were concentrated by vacuum centrifugation to near dryness and desalted using C18 stage-tip columns. Phosphopeptides were enriched using titanium dioxide (TiO_2_) before LC-MS/MS analysis.

Liquid chromatography–mass spectrometry (LC-MS/MS) was performed using an EASY-nLC 1000 system coupled online to a Fusion Lumos mass spectrometer (Thermo Fisher Scientific). Peptide separation was carried out using a 75 μm × 20 cm in-house packed column (ReproSil-Pur C18-AQ, 3 μm; Dr Maisch GmbH) with Buffer A (0.1% formic acid in water) and Buffer B (0.1% formic acid in acetonitrile) as mobile phases. Peptides were eluted with a linear gradient of 3 to 30% Buffer B over 50 min, followed by 30 to 90% Buffer B over 10 min, at a flow rate of 300 nl/min.

For the analysis of acetylation and phosphorylation modifications, the mass spectrometer was operated in data-dependent acquisition (DDA) mode. For global protein profiling, data-independent acquisition (DIA) mode was employed. In DDA mode, full MS scans were acquired in the Orbitrap mass analyzer over a range of 300 to 1500 m/z at a resolution of 60,000 (at m/z 200). The top 15 most intense precursor ions (charge states 2–6) were selected for fragmentation using higher-energy collisional dissociation (HCD) with a normalized collision energy of 35 and an isolation window of 1.6 m/z. MS/MS spectra were acquired in the Orbitrap at a resolution of 15,000. Automatic gain control (AGC) targets were set to 1e6 for MS scans and 5e4 for MS/MS scans, with a maximum injection time of 50 ms for both.

In DIA mode, MS1 scans were collected from 350 to 1400 m/z at a resolution of 120,000. Precursors from 350 to 975 m/z were isolated using 45 variable windows of 14 m/z width with 1 m/z overlap and fragmented using HCD (normalized collision energy 35). MS/MS spectra were acquired in the Orbitrap at 15,000 resolution, with an AGC target of 1e6 for both MS1 and MS2 scans.

DDA raw files were processed using MaxQuant version 1.6.17.0 (Max Planck Institute). Carbamidomethylation of cysteine was set as a fixed modification. Variable modifications included methionine oxidation, protein N-terminal acetylation, lysine acetylation, and phosphorylation of serine, threonine, and tyrosine. Precursor and fragment ion mass tolerances were set to 7 ppm and 20 ppm, respectively, allowing up to two missed cleavages. Both peptide and protein identifications were filtered at a 1% false discovery rate (FDR) using a decoy database of reversed protein sequences. Identified phosphopeptides and acetylated peptides were manually inspected to confirm modification site localization.

DIA raw files were processed using DIA-NN v1.8 with one missed cleavage allowed. Protein and peptide identifications were filtered at a 1% FDR, and default mass tolerance settings were applied. Carbamidomethylation of cysteine was set as a fixed modification, and methionine oxidation as a variable modification. For both DDA and DIA datasets, protein identification was performed against the UniProt human protein database (downloaded August 7, 2021).

#### Hsp90 MIND-S model

The MIND-S model, a transformer-based framework for post-translational modification (PTM) prediction, was fine-tuned using curated HSP90 sequences. The dataset consisted of six input sequences: the alpha and beta isoforms of HSP90 in their wild-type (WT) forms, as well as variants carrying two specific mutations, lysine-to-glutamine (K→Q), and lysine-to-arginine (K→R). These sequences were selected to capture potential PTM patterns across isoforms and mutation contexts. Each sequence was between 610 and 732 amino acids in length and was selected for its biological relevance in studying PTMs on lysine residues, especially in relation to acetylation, methylation, and ubiquitination. These variants allow evaluation of mutation-driven PTM changes in both sequence and structural context.

##### Preprocessing

Each sequence was tokenized to prepare it for input into the MIND-S model. Special tokens marking the start and end of each sequence were appended, and all sequences were padded to ensure a uniform length of 514 amino acids. Positional information was integrated into the input representation using sinusoidal positional encodings, which were combined with the tokenized sequences. Tokenization used the amino acid vocabulary defined in the MIND implementation. The sinusoidal embeddings were computed in 128 dimensions and applied consistently across sequences. Input transformation was handled through a dedicated Sequence Transformer class, which wrapped the original utility functions and ensured the sequences were converted to appropriate NumPy arrays before model ingestion. The output of the model was configured to predict the expected PTM types for all potential modification sites within the sequences. The model supported prediction for 13 PTM types, including phosphorylation, methylation (K and R), acetylation, ubiquitination, SUMOylation, and glycosylation.

The target data, representing site-PTM pairs, were transformed into a matrix format suitable for model training. Instead of a binary matrix (0 or 1) indicating PTM presence, a continuous score between 0 and 1 was introduced for training outcomes. This score allows for a more nuanced representation of PTM likelihoods and accounts for subtle variations between WT and mutant sequences.

##### Fine-tuning procedure

Fine-tuning was performed using pretrained MIND-S models. To retain the generalizable features learned during the initial training of the MIND-S model, the weights of all layers were frozen, except for the final encoding layer and the last dense layer. The training process was carried out over 500 epochs, using the Adam optimizer with a learning rate set to 1 × 10^−4^. Binary cross-entropy was used as the loss function, and model performance was evaluated based on binary accuracy. Training was implemented using TensorFlow/Keras with manual inspection of training dynamics through console output. To enhance robustness and ensure reliable predictions, the fine-tuning procedure was repeated for 15 models using the original 15 pretrained models as a baseline. Each fine-tuned model was adapted to the specific input sequences while maintaining the overall structure of the MIND-S architecture. The final predictions were derived by combining the results across all models. This ensemble approach used PTM-type–specific weights derived from the area under the precision-recall curve (PR-AUC) of the original pretrained models, enhancing the contribution of more reliable base models for each PTM. This helped compensate for the small dataset size and led to more stable and consistent predictions across PTM types.

##### PTM score calculation

To better model differences in PTM type prediction across mutations and wild-type sequences, the PTM scores were determined based on experimental mass spectrometry (MS) data. The wild-type (WT) score was fixed at 0.75, ensuring that it remains within the positive PTM prediction class (>0.5) while allowing room for both increases and decreases in modification likelihood. For mutated sequences, the log(mutation/WT) value was used as a baseline (x) to compute scores. These values were derived from observed intensity changes in the MS data and were clipped to a reasonable range to avoid instabilities from extreme fold-changes. To normalize the scores within the [0.1] range while maintaining interpretability, the transformation applied was:

$$score=\left(\frac{x+1}{2}\right)+0.25$$

This transformation ensures that log-ratio-based changes are adjusted to fit the expected PTM likelihood scale. It maps a log value of 0 (indicating no change between WT and mutation) to 0.75, aligning it with the WT score. The chosen formula is tailored to our dataset, ensuring that all log values remain within the 0 to 1 range without exceeding these bounds. By integrating these refined PTM probabilities, the model can more effectively capture PTM dynamics under different mutational contexts, ultimately leading to more accurate and biologically meaningful predictions.

### Protein extraction from mammalian cells

Protein extraction from mammalian cells was carried out using methods previously described (34). Adherent cells were washed with ice cold PBS and lysed with 200 μl lysis buffer (20 mM Tris-HCl (pH 7.4), 100 mM NaCl, 1 mM MgCl_2_, 0.1% NP40, protease inhibitor cocktail (Roche), and PhosSTOP (Roche)). Cells were briefly sonicated and centrifuged at 4 °C, 10,000*g* for 8 min to pellet cell debris. The supernatant (cell lysate) was transferred to a fresh tube and stored at −80 °C or used in downstream assays.

### Pulldown and immunoblotting

For biotinylated-GB pulldown mammalian cell lysate was collected and incubated with Biotin-GB (0.1 μM or 0.5 μM) for 30 min. Streptavidin beads were then added and incubated for an additional 30 min. Protein bound to beads was washed with fresh extraction buffer 4 times and eluted in 5× Laemmli buffer. Proteins were eluted from beads were run on SDS-PAGE gel for immunoblot or mass spectrometry analysis. For ATP pulldown cell lysate was collected and incubated with ATP-agarose for 2 h. Protein bound to beads was washed with fresh extraction buffer 4 times and eluted in 5× Laemmli buffer. Proteins were eluted from beads and run on SDS-PAGE gel for immunoblot analysis. Co-precipitated proteins and inputs were detected by immunoblotting with antibodies recognizing FLAG (ThermoFisher Scientific), GAPDH, HDAC1, HDAC2, HDAC3, HDAC4, HDAC5, HDAC6, HDAC7, HDAC8, HDAC11 (Cell Signalling), HDAC9, HDAC10 (Abcam), and Hsp90 (Enzo). Secondary antibodies raised against mouse, rabbit, and rat (Cell Signaling) were also used.

### Quantification and statistical analysis

Densitometry was performed using Photoshop v.23.5.1 to quantify immunoblot band signal intensity. All statistics were performed using GraphPad Prism version 9.2.0 for macOS (GraphPad Software, www.graphpad.com).

#### Preparation of figures

Some Figure panels were prepared using BioRender software (https://biorender.com/).

#### Software and algorithms

Biorender (https://biorender.com/), Cytoscape (V3.10.2), and ChimeraX (V1.8rc202405202242 (35), Interactive Peptide Spectral Annotator (36), and Graphpad Prism (V9.2.0 for macOS) were all used.

#### Data and code availability

The mass spectrometry-proteomics data have been deposited to the ProteomeXchange Consortium *via* the PRIDE partner repository with the identifier: PXD061927 (32) and are publicly available as of the date of publication. The reviewers may access the data by Log in to the PRIDE website using the following details: Project accession: PXD061927; Token: ynzKAIsTVoaC. The fine-tuning of the MIND-S model for HSP90 post-translational modification (PTM) prediction was performed within a Jupyter Notebook, utilizing the publicly available MIND-S model implementation. All original code has been deposited at Mendeley Data Digital Commons and is publicly available at https://doi.org/10.17632/dk8yb2prrw.1 as of the date of publication. The original MIND-S implementation can be accessed at https://github.com/yuyanislearning/MIND.

A video abstract is available at https://doi.org/10.1016/j.jbc.2025.110519#mmc6.

## Supporting information

This article contains supporting information.

## Conflict of interest

The authors declare that they have no conflicts of interest with the contents of this article.
