# Supplementary material for: Integrating deep learning for post-translational modifications crosstalk on Hsp90 and drug binding[image]
Source: J Biol Chem. 2025 Jul 25;301(9):110519. doi: 10.1016/j.jbc.2025.110519 (PMC12398796; doi:10.1016/j.jbc.2025.110519)
Supplement: Supplementary Material [file mmc1.pdf]

Integrating deep learning for post-translational modifications crosstalk on Hsp90 and drug binding

Jennifer A. Heritz[1,2,3,6], Katherine A. Meluni[1,2,3,6], Sarah J. Backe[1,3], Sara J. Cayaban[1,2,3], Laura A. Wengert[1,2,3], Meik Kunz[4], Mark R. Woodford[1,2,3], Dimitra Bourboulia[1,2,3], Mehdi Mollapour[1,2,3,5,†]

[1] Department of Urology, SUNY Upstate Medical University, Syracuse, NY, 13210, USA

[2] Department of Biochemistry and Molecular Biology, SUNY Upstate Medical University, Syracuse, NY, 13210, USA

[3] Upstate Cancer Center, SUNY Upstate Medical University, Syracuse, NY, 13210, USA

[4] The Bioinformatics CRO, Sanford Florida, 32771 USA.

[5] Lead Contact

[6] These authors contributed equally

[†] Correspondence to mollapom@upstate.edu

Supporting Information

This article contains supporting information.

Table S1. Hsp90-bio-GB pulldown interactome mass-spectrometry results

Table S2. Hsp90-bio-GB pulldown acetylation mass-spectrometry results

Table S3. Hsp90-bio-GB pulldown phosphorylation mass-spectrometry results

Table S4. AI Model raw prediction values

Table S5. Western blot quantification values

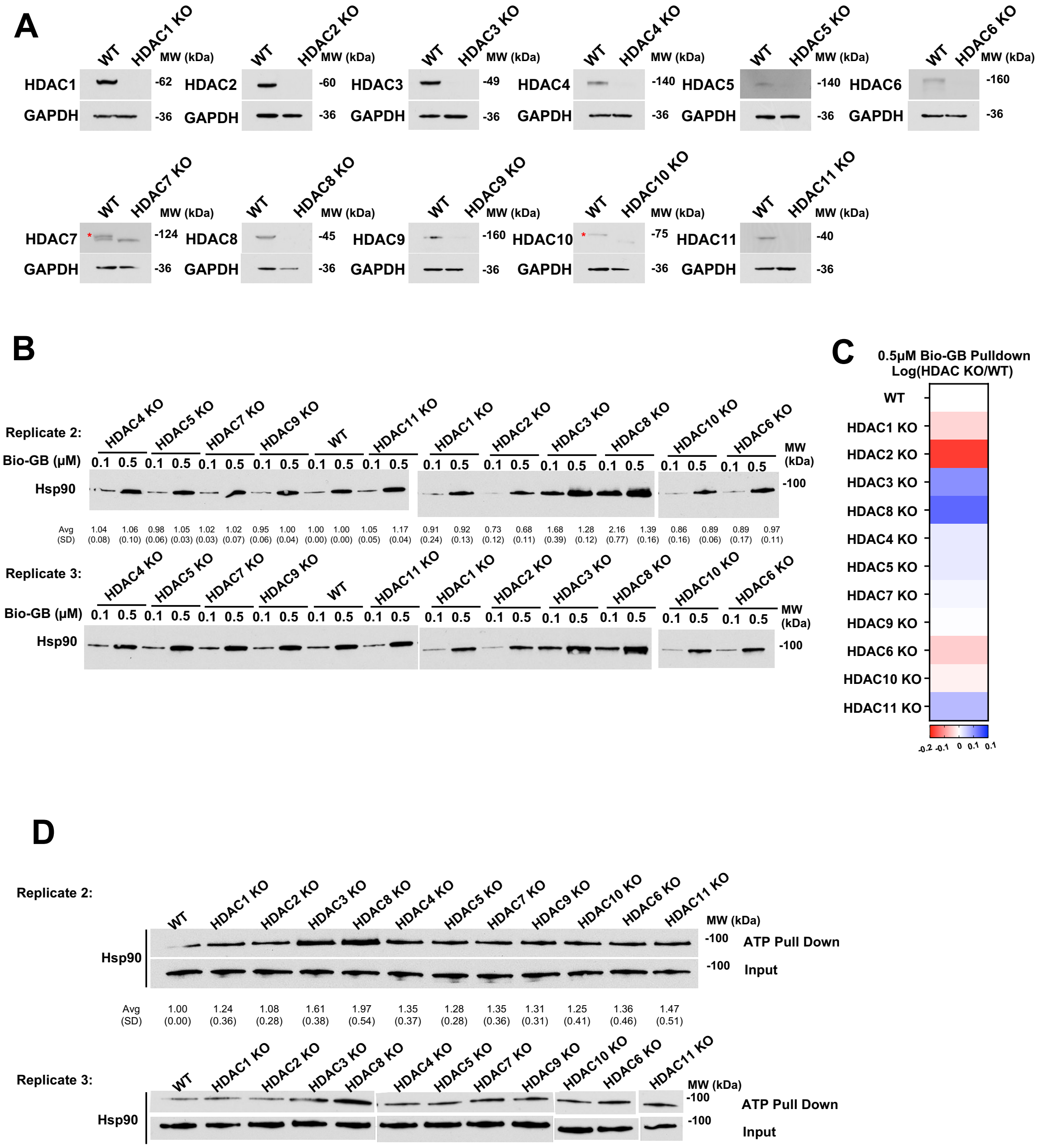

Figure S1. Loss of *HDAC3* or *HDAC8* impacts Hsp90 binding to drug and ATP. A) HAP1 WT or HAP1 HDAC KO cell lysate was collected and immunoblotted with the indicated HDAC specific antibody to confirm KO. Red asterisks indicate the relevant protein band. B) HAP1 WT or HDAC1-11 KO cell lysate were challenged with 0.1 µM or 0.5 µM Biotin-GB (Bio-GB). Hsp90 binding to Bio-GB was assessed by immunoblotting. Average and standard deviation (SD) values indicate ratios of sample pulldown/wild type (WT) pulldown of 3 independent experiments; see also Figure 1B. Sample pulldown and WT pulldown values were normalized to respective input values; see Figure 1E, S1D. C) Heatmap of

densitometry values from **Figure 1A,B, S1B** quantifying Hsp90 binding to 0.5 μM Biotin-GB. Data displayed is log(fold-change) intensity of the Hsp90 bound to 0.5 μM Biotin-GB in each HAP1 HDAC KO relative to Hsp90 bound to 0.5 μM Biotin-GB in HAP1 WT. Red shading indicates decreased binding of Hsp90 to Biotin-GB whereas blue shading indicates increased binding of Hsp90 to Biotin-GB. To ensure accurate quantifications, Hsp90 pulldown values were normalized to Hsp90 input values for each sample. D) HAP1 WT or HAP1 HDAC KO cell lysate was collected and incubated with ATP agarose beads for 2 hours. Proteins were eluted from beads and run on SDS-PAGE gel for immunoblot analysis. Average and standard deviation (SD) values indicate ratios of sample pulldown/wild type (WT) pulldown of 3 independent experiments; see also Figure 1E. Sample pulldown and WT pulldown values were normalized to respective input values; see Figure 1E.

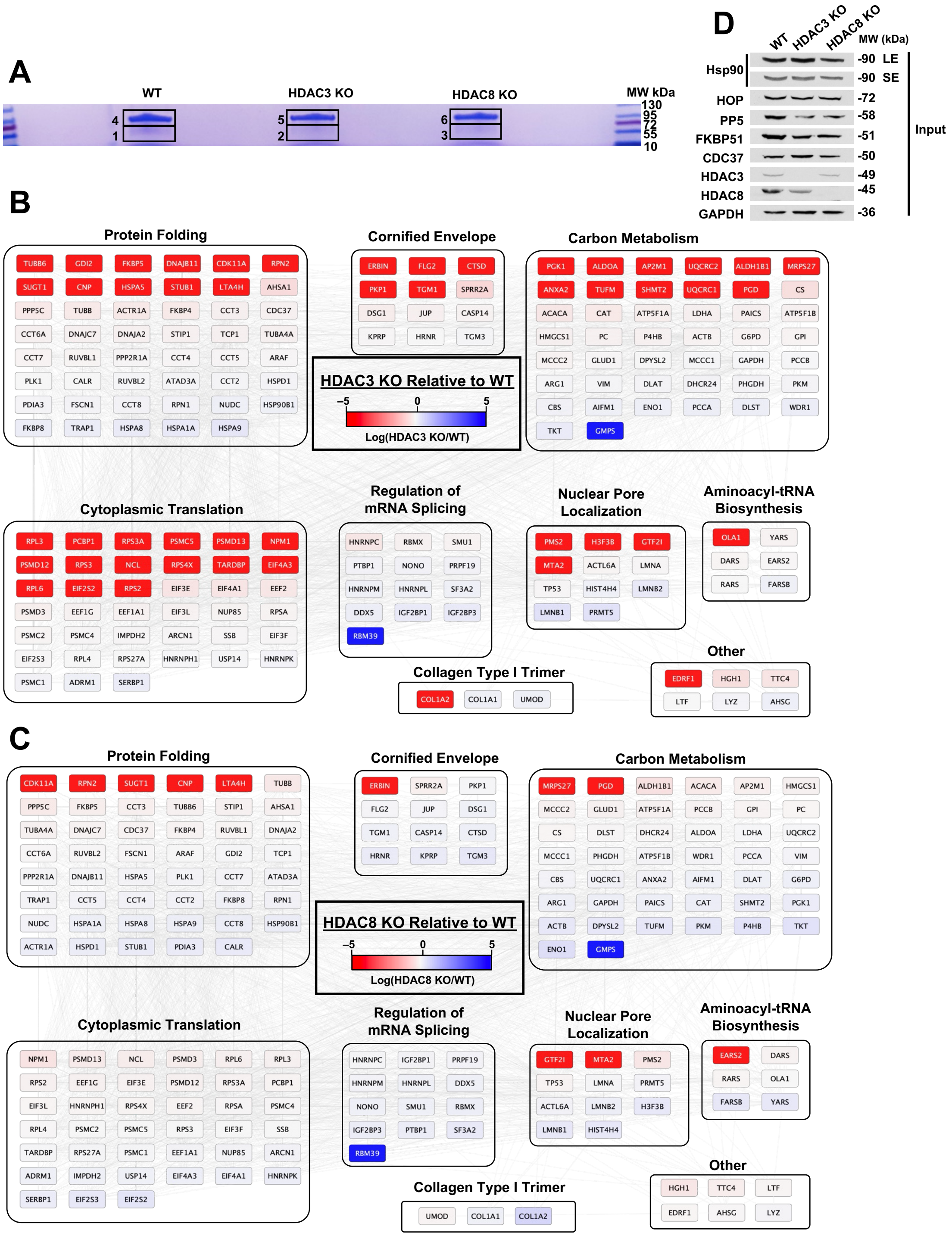

Figure S2. Deletion of *HDAC3* or *HDAC8* differentially dictates global interactome of Hsp90 bound to drug. A) Coomassie stained gel of Hsp90-bio-GB complex samples for mass spectrometry. Boxes 1, 2 and 3 represent the excised bands used for interactome analysis. Boxes 3, 5 and 6 represent the

excised bands sent for Hsp90-PTM analysis. See also Figure 2-4. B) Proteins identified by mass spectrometry in the Hsp90-bio-GB complex of HAP1 WT compared to *HDAC3* KO were grouped based on biological process gene ontology from string.db. Red indicates proteins with decreased interaction with Hsp90-bio-GB complex in *HDAC3* KO, blue indicates proteins with increased interaction with Hsp90-bio-GB complex in *HDAC3* KO relative to WT HAP1. C) Proteins identified by mass spectrometry in the Hsp90-bio-GB complex of HAP1 WT compared to *HDAC8* KO were grouped based on biological process gene ontology from string.db. Red indicates proteins with decreased interaction with Hsp90-bio-GB complex in *HDAC8* KO, blue indicates proteins with increased interaction with Hsp90-bio-GB complex in *HDAC8* KO relative to WT HAP1. D) HAP1 WT, *HDAC3* KO, and *HDAC8* KO cell lysate was collected and immunoblotted with antibodies for proteins identified to have decreased interaction with Hsp90-bio-GB complex.

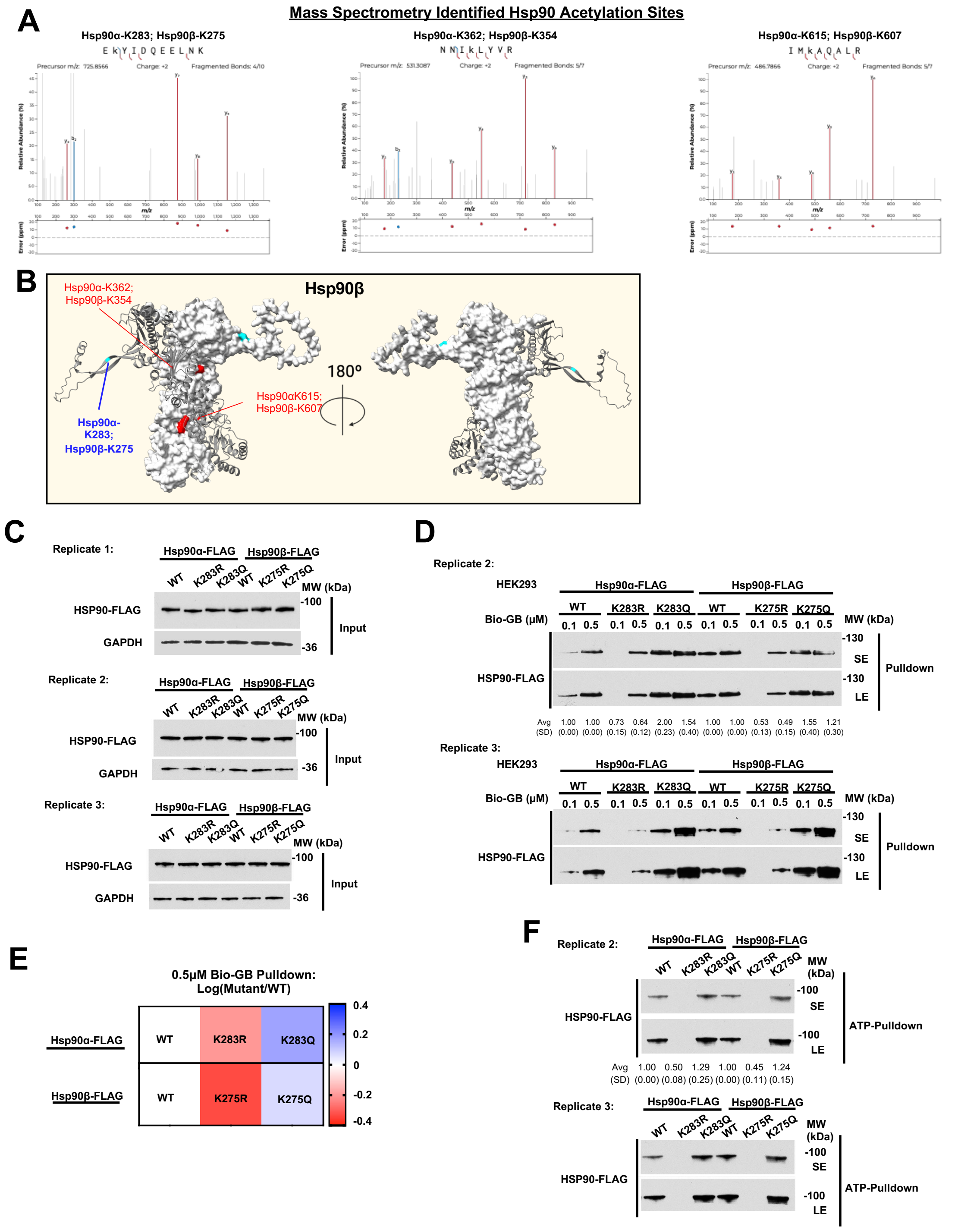

Figure S3. Hyperacetylation of Hsp90α-K283 and Hsp90β-K275 enhances drug binding. A) Mass spectrometry traces of acetylated Hsp90 bound to drug isolated from HAP1 WT, *HDAC3 KO*, or *HDAC8 KO* displayed using the Interactive Peptide Spectral Annotator. See also Figures 3B-C. B) Hsp90 acetylated lysine sites highlighted on Hsp90β Alphafold structure (amino acids 14-686 shown).

Residues that could not be discerned to be either Hsp90α or Hsp90β are separated by a semi-colon. Residues shown in red are hypoacetylated. Residues shown in blue/cyan are hyperacetylated. C) HEK293 cells transiently expressing WT-Hsp90α, K283R-Hsp90α, or K283Q-Hsp90α; WT-Hsp90β, K275R-Hsp90β, or K275Q-Hsp90β. D) Cell lysate from HEK293 cells transiently expressing Hsp90 constructs was collected and incubated with Biotin-GB (0.1 μM or 0.5 μM) for 30 minutes. Streptavidin beads were then added and incubated for an additional 30 minutes. Proteins were eluted from beads and run on SDS-PAGE gel for immunoblot analysis. Average and standard deviation (SD) values indicate ratios of sample pulldown/wild type (WT) pulldown of 3 independent experiments; see also Figure 3D. Sample pulldown and WT pulldown values were normalized to respective input values; see Figure S3C. E) Heatmap of densitometry values from **Figure 3D, S3C,D** quantifying WT-Hsp90-FLAG or acetylation-blocking (R) or acetylation-mimicking (Q) mutants binding to 0.5 μM Biotin-GB. Hsp90 constructs were transiently expressed in HEK293 cells. Cell lysate was incubated with 0.5 μM Biotin-GB for 30 minutes. Streptavidin beads were then added and incubated for an additional 30 minutes. Hsp90 binding to GB was assessed by immunoblot. Data displayed is log(fold-change) intensity of each Hsp90-FLAG construct bound to 0.5 μM Biotin-GB relative to WT-Hsp90-FLAG bound to 0.5 μM Biotin-GB. Red shading indicates decreased binding of the Hsp90 construct to Biotin-GB whereas blue shading indicates increased binding of the Hsp90 construct to Biotin-GB. To ensure accurate quantifications, Hsp90-FLAG pulldown values were normalized to Hsp90-FLAG input values for each sample. F) Cell lysate from HEK293 cells transiently expressing WT-Hsp90-FLAG or acetylation-blocking (R) or acetylation-mimicking (Q) mutants was collected and incubated with ATP-agarose for 2 hours. Proteins were eluted from beads and run on SDS-PAGE gel for immunoblot analysis. Average and standard deviation (SD) values indicate ratios of sample pulldown/wild type (WT) pulldown of 3 independent experiments; see also Figure 3F. Sample pulldown and WT pulldown values were normalized to respective input values; see Figure S3C.

Mass Spectrometry Identified Hsp90 Phosphorylation Sites

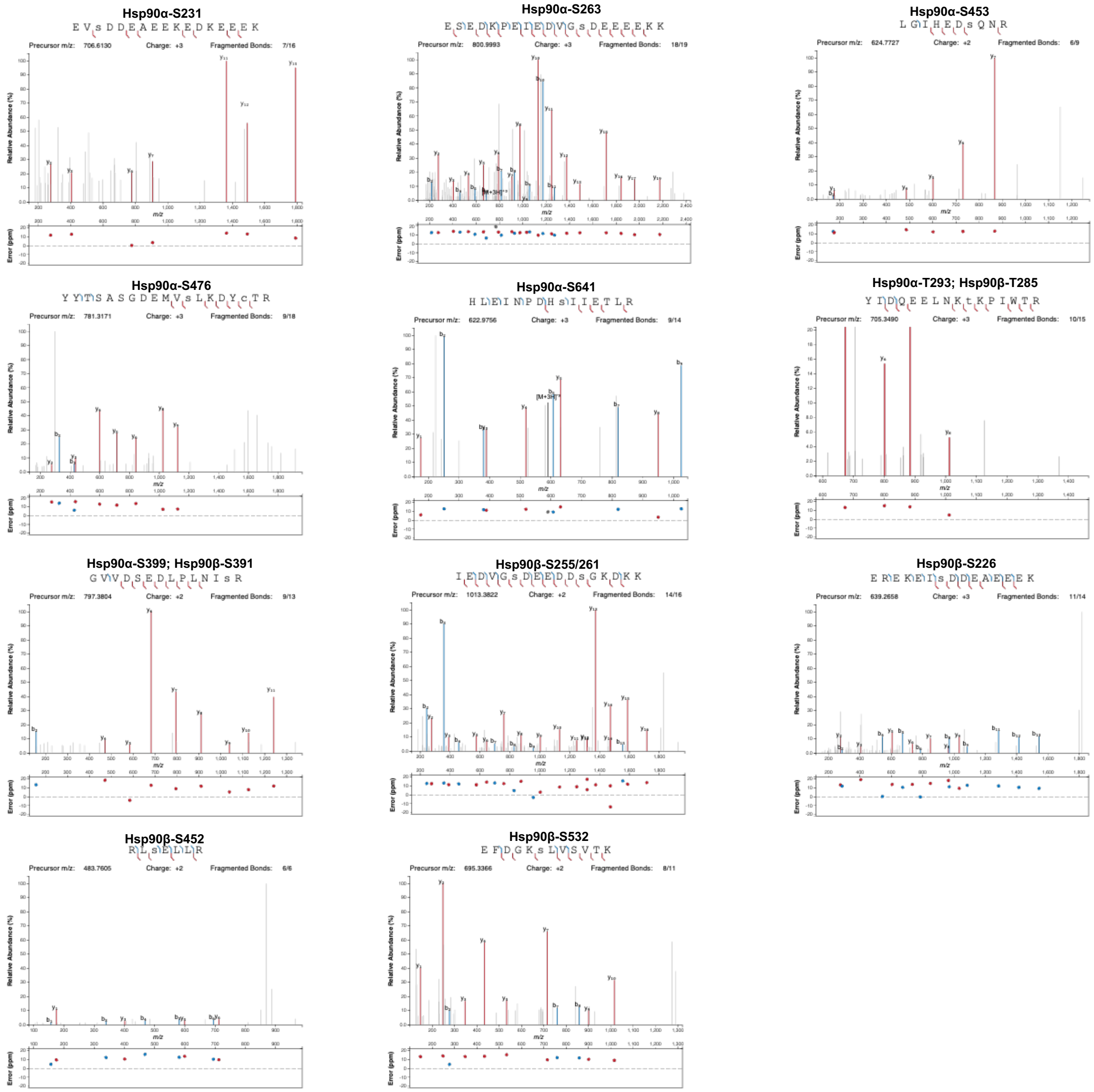

Figure S4. Predicting PTM crosstalk of Hsp90 using a deep learning AI model. Mass spectrometry traces of phosphorylated Hsp90 bound to drug isolated from HAP1 WT, *HDAC3 KO*, or *HDAC8 KO* displayed using the Interactive Peptide Spectral Annotator.
